# Cherenkov emission–based quality assurance for linear accelerators

**DOI:** 10.1093/jrr/rraf072

**Published:** 2026-01-13

**Authors:** Hiroyuki Okamoto, Fuma Tojo, Kazuyoshi Kurita, Miyuki Murata, Masataka Ueda, Aya Shimoike, Yuka Kondou, Yukio Fujita, Yuna Nakajima, Satoshi Nakamura, Kotaro Iijima, Takahito Chiba, Hiroki Nakayama, Tetsu Nakaichi, Hiroshi Igaki

**Affiliations:** Section of Radiation Safety and Quality Assurance, National Cancer Center Hospital, 5-1-1 Tsukiji, Chuo-ku, Tokyo 104-0045, Japan; College of Science Department of Physics, Rikkyo University, 3-34-1 Nishiikebukuro, Toshima-ku, Tokyo 171-8501, Japan; College of Science Department of Physics, Rikkyo University, 3-34-1 Nishiikebukuro, Toshima-ku, Tokyo 171-8501, Japan; College of Science Department of Physics, Rikkyo University, 3-34-1 Nishiikebukuro, Toshima-ku, Tokyo 171-8501, Japan; Department of Radiological Technology, National Cancer Center Hospital, 5-1-1 Tsukiji, Chuo-ku, Tokyo 104-0045, Japan; Department of Radiological Technology, National Cancer Center Hospital, 5-1-1 Tsukiji, Chuo-ku, Tokyo 104-0045, Japan; Department of Radiological Technology, National Cancer Center Hospital, 5-1-1 Tsukiji, Chuo-ku, Tokyo 104-0045, Japan; Department of Radiological Technology, National Cancer Center Hospital, 5-1-1 Tsukiji, Chuo-ku, Tokyo 104-0045, Japan; Department of Radiological Sciences, Komazawa University, 1-23-1 Komazawa, Setagaya-ku, Tokyo 154-8525, Japan; Department of Radiological Sciences, Komazawa University, 1-23-1 Komazawa, Setagaya-ku, Tokyo 154-8525, Japan; Section of Radiation Safety and Quality Assurance, National Cancer Center Hospital, 5-1-1 Tsukiji, Chuo-ku, Tokyo 104-0045, Japan; Department of Radiation Oncology, Juntendo University Graduate School of Medicine, 2-1-1 Hongo, Bunkyo-ku, Tokyo 113-8421, Japan; Section of Radiation Safety and Quality Assurance, National Cancer Center Hospital, 5-1-1 Tsukiji, Chuo-ku, Tokyo 104-0045, Japan; Section of Radiation Safety and Quality Assurance, National Cancer Center Hospital, 5-1-1 Tsukiji, Chuo-ku, Tokyo 104-0045, Japan; Section of Radiation Safety and Quality Assurance, National Cancer Center Hospital, 5-1-1 Tsukiji, Chuo-ku, Tokyo 104-0045, Japan; Department of Radiation Oncology, National Cancer Center Hospital, 5-1-1 Tsukiji, Chuo-ku, Tokyo 104-0045, Japan

**Keywords:** Cherenkov emission, IGRT QA, machine QA, radiotherapy

## Abstract

When electrons exceed the speed of light in a medium, they emit low-intensity visible light, known as Cherenkov emission (CE). This study proposes a novel CE-based quality assurance (QA) test for linear accelerators. A CE-based QA (C-QA) phantom incorporating a mock tumor and four CE observation plates (top, bottom, left, and right) was developed. After tumor-based alignment using cone-beam computed tomography (CBCT), lateral and posterior fields were used for irradiation. A C-Dose camera was employed to measure the treatment position, gantry angle, photon energy (*TPR*_20,10_), and CE counts for both fields. The treatment position and *TPR*_20,10_ were determined by analyzing the changes in the CE profile, while the gantry angle was calculated based on the tilt between the entry and exit field positions. Confidence limits were evaluated over a three-month period, during which long-term testing demonstrated favorable results. The standard deviations (σ) for CBCT-based positional accuracy and gantry angle were within ±1 mm in all three directions and within 1°, respectively. The mean ± σ for *TPR*_20,10_ was 0.631 ± 0.004, closely matching the 0.629 measured using an ionization chamber. Detected CE counts exhibited a higher variation (σ = 2.7%). CE-based QA appears to be an effective and reliable method for radiotherapy. Treatment position could be directly measured without conventional dosimetric devices, while CE imaging simultaneously evaluated positional accuracy, gantry angle, and photon energy (*TPR*_20,10_). However, accurate assessment of linear accelerator dose output remains a challenge, and the quantification of CE counts requires further investigation.

## INTRODUCTION

Cherenkov emission (CE) occurs when electrons travel faster than the speed of light in a medium, producing visible light of low intensity in a specific direction [1]. The intensity of CE is much lower than the ambient light in the treatment room. Thus, it cannot be observed with the naked eye and requires a dedicated high-sensitivity camera, such as an intensified charge-coupled device (ICCD) camera [2]. In megavoltage photon radiotherapy, high-energy scattered electrons are generated in media such as water, quality assurance (QA) phantoms, and human tissues (including skin and muscle), making it possible to image CE using a dedicated camera. In radiotherapy, measuring CE near the surface of a medium enables evaluation of daily treatment accuracy and facilitates QA for treatment delivery [3–5]. Cherenkov imaging, or ‘Cherenkoscopy’ does not require conventional measurement tools such as dosimeters, allowing direct assessment of dosimetric information from the treatment site. It offers advantages such as being noninvasive, comfortable for patients, and efficient for in vivo dosimetry. The first Cherenkov images in breast radiotherapy patients were reported in 2014, enabling real-time visualization of surface skin dose [5]. Recently, numerous studies have investigated its application in QA across various treatment modalities and purposes [6–14]. However, investigating on Cherenkov-based image-guided radiotherapy (IGRT) QA are limited.

This study aims to propose a novel concept of a Cherenkov-based end-to-end (E2E) phantom for image-guided radiotherapy (IGRT) QA testing. This approach eliminates the need for conventional dosimeters, such as radiochromic films [15, 16], and directly assesses positional treatment accuracy through CE imaging.

## MATERIAL AND METHODS

### Cherenkov emission-based QA phantom

**Fig. 1:**
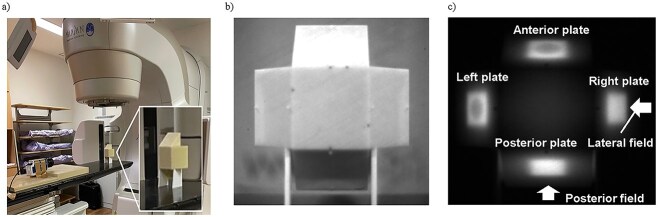
(a) Measurement setup with the C-Dose camera. (b) Background image of the C-QA phantom. (c) CE observed during irradiation from lateral and posterior fields (gantry angles 90° and 180°, respectively).

A CE-based QA phantom (C-QA) for IGRT was developed, as shown in Fig. 1 and Supplementary Fig. 1. A mock tumor, 2 cm in diameter and made of tungsten, was placed at the center of the phantom. The phantom body was constructed from ABS resin (Taisei Medical, Kyoto, Japan). Four slanted plates were positioned on the top, bottom, left, and right sides of the phantom to enable the C-Dose camera (DoseOptics, Lebanon, NH, USA) to capture CE from their surfaces. As shown in Fig. 1, the angle of the slanted plate was set to 30 degrees. A larger angle would allow a wider Cherenkov light observation surface, but would also increase the size of the phantom. Considering both factors, we selected 30 degrees. As described later, the spatial resolution in this setup corresponds to 0.1 mm, ensuring adequate precision. The C-Dose camera was positioned 1 m inferior to the isocenter for all tests (Fig. 1a), equipped with a 50 mm single-focus lens (Ai AF Nikkor 50 mm F1.8D, Nikon, Tokyo, Japan). CT scans were performed using a Canon Aquilion One CT scanner (Canon, Tokyo, Japan). The IGRT QA treatment plan was prepared using Varian Eclipse (Varian Medical Systems, Palo Alto, CA, USA). A Varian TrueBeam linear accelerator was used for irradiation, delivering 6 MV flattening filter-free (FFF) orthogonal fields (90° and 180° gantry angles) with a field size of 3 × 3 cm^2^ and 800 monitor units (MU). After tumor-based alignment using cone-beam CT (CBCT), the C-QA phantom was imaged during irradiation with the C-Dose camera. The resolution of the C-Dose camera is 1200 $\times$ 1600 (vertical $\times$ horizontal), which corresponds to a spatial resolution of about 0.1 mm.

### Cherenkov camera

Accurate measurements using camera-based imaging dosimetry require multiple corrections, including background noise, sensor noise, stray radiation, vignetting, and lens distortion. The C-Dose camera employs a scintillator-based gating technique, where the detection of stray radiation in the scintillator can trigger the gating, enabling the imaging of extremely low-intensity CE. The scintillator is integrated into the camera, and dark images (captured with the lens cap on) are acquired to allow for correction. Background images (acquired without radiation) are collected outside the gating sequence during image processing. However, in this study, vignetting and lens distortion corrections were not applied, as positional accuracy was evaluated using an analytical method described later.

### Analysis

A model for calculating treatment positions was developed based on the relationships between changes in the CE profile and the positions of the C-QA phantom. Predefined phantom displacements were applied in three directions: right–left (RL, where right indicates positive displacement), superior–inferior (SI, where superior indicates positive displacement), and anterior–posterior (AP, where anterior indicates positive displacement). The predetermined displacements ranged from −2 to 2 mm in each direction, and the C-QA phantom was shifted accordingly by remotely adjusting the patient couch position. For each field, 800 MU were delivered, and the C-Dose camera imaged the C-QA phantom during irradiation. The model for calculating treatment positions was established using the predefined displacements and the ratio of the two peaks in the CE profile at the left and the anterior plate. A second-order polynomial function was applied to the data, as it yielded a higher coefficient of determination compared to a first-order fit. Four test patterns (Table 1) were independently conducted on different dates to evaluate the model’s accuracy. Statistical analyses were performed using MATLAB 2020a (MathWorks, Natick, MA, USA).

**Table 1:** Tests for evaluating treatment position accuracy using the C-QA phantom

	Test1			Test 2			Test 3			Test 4		
Direction	RL	SI	AP	RL	SI	AP	RL	SI	AP	RL	SI	AP
Nominal	1	−1	1	−2	2	−2	0.5	−0.5	0.5	3	−3	3
Test	1.06	−1.08	1.05	−2.10	2.03	−2.04	0.56	−0.63	0.55	3.16	−2.7	3.32
Diff	0.06	−0.08	0.05	−0.1	0.03	−0.04	0.06	−0.13	0.05	0.16	0.30	0.32

C-QA: Cherenkov emission-based QA. RL: right–left; SI: superior–inferior; AP: anterior–posterior; Diff: difference. Data are presented as mm.

### Gantry angle, energy characteristics, repeatability, and Cherenkov emission

The Supplementary Fig. 2 shows the method for calculating the gantry angle for the posterior field. In principle, for the posterior field, the gantry angle is calculated from the offset from the center of the lower plate, the offset from the center of the upper plate, and the vertical length at that position, using the arctangent. For the lateral field, the calculation is performed using the RL plates, and the same calculation method is applied. Additionally, the energy characteristics of CE were investigated by analyzing three parameters: CE on the phantom surface at the right plate, CE at the left plate produced by attenuated radiation, and the ratio of CE on the paired RL plates (denoted as *R*_en_), calculated using the following equation:


(1) \begin{equation*}\,\,\,\,\,\,\,\,\,\,\,\,\,\,\,\,\,\,\,\,\,\,\,\,\,\,\,\,\,\,\,\,\,\,\,\,\,\,\,\,\,\,\,\,\,\,\,\,\,\,\,\, {R}_{\mathrm{en}}=\frac{C_{\mathrm{L}}}{C_{\mathrm{R}}} \end{equation*}

*C*_R_ and *C*_L_ represent the detected Cherenkov counts within the region of interest (ROI) of about 1 × 1 cm^2^ at the center of the radiation field on the right and left plates, respectively. The relative positions between C-Dose camera and C-QA phantom slightly varies among tests. However, in-house software identifies the phantom body on the obtained image, and it allows to automatically define the ROIs on the four plates at approximately the same location. All photon energies were characterized using *TPR*₂₀,₁₀ measurements obtained with the PTW Semiflex Ionization Chamber 31 010 (PTW-Freiburg GmbH, Germany). *TPR*₂₀,_10_ values were calculated by applying a regression curve to the measured data. The photon energies available on the Varian TrueBeam were 4X, 6X, 10X, 15X, 6X FFF, and 10X FFF. Furthermore, repeatability (*n* = 5) was assessed in terms of treatment position after tumor-based alignment using CBCT (i.e. ideally zero displacement), gantry angle, *TPR*₂₀,₁₀, and detected Cherenkov counts within about 1 × 1 cm^2^ ROIs on the beam entry plates. These five tests were conducted on separate days.

### Ambient treatment room light

We evaluated whether variations in ambient treatment room light affected the CE profile. Noise within a 1 × 1 cm^2^ region for a 10 × 10 cm^2^ field size at the surface of a slab water-equivalent phantom (Tough Water Phantom, Kyoto Kagaku, Kyoto, Japan), with a 10 cm thick backscatter phantom, was measured using the C-Dose camera under three ambient light levels (minimum, medium, and maximum), adjusted via the dimmer switch in the treatment room. The medium light level was approximately the same as that used during patient setup. The light intensity was measured using a digital illuminometer (IM-600 M, Topcon Technohouse Corporation, Tokyo, Japan).

### Long-term tests

Long-term tests for positional treatment accuracy using CBCT-based image guidance, gantry angle, *TPR*₂₀,₁₀, and detected Cherenkov counts were conducted over approximately three months. A total of seventeen tests were performed, averaging one measurement per week. For efficiency, the irradiated MU were reduced to 300 MU, while all other treatment parameters remained the same as in previous tests. Gantry angle, *TPR*₂₀,₁₀, detected Cherenkov counts, and positional accuracy were evaluated in each measurement. *TPR*₂₀,₁₀ was calculated using the regression curve obtained from the previously described energy characteristics test. The detected Cherenkov counts were expected to monitor the delivered MU and were normalized to the first measurement for evaluation. Additionally, the normalized data were compared with X-ray output measurements obtained using a QUICKCHECK webline (PTW-Freiburg, Germany). The resulting trend data were also used to determine confidence limits, calculated as 1.96 ± σ (standard deviation) in terms of measurement repeatability.

## RESULTS

**Fig. 2:**
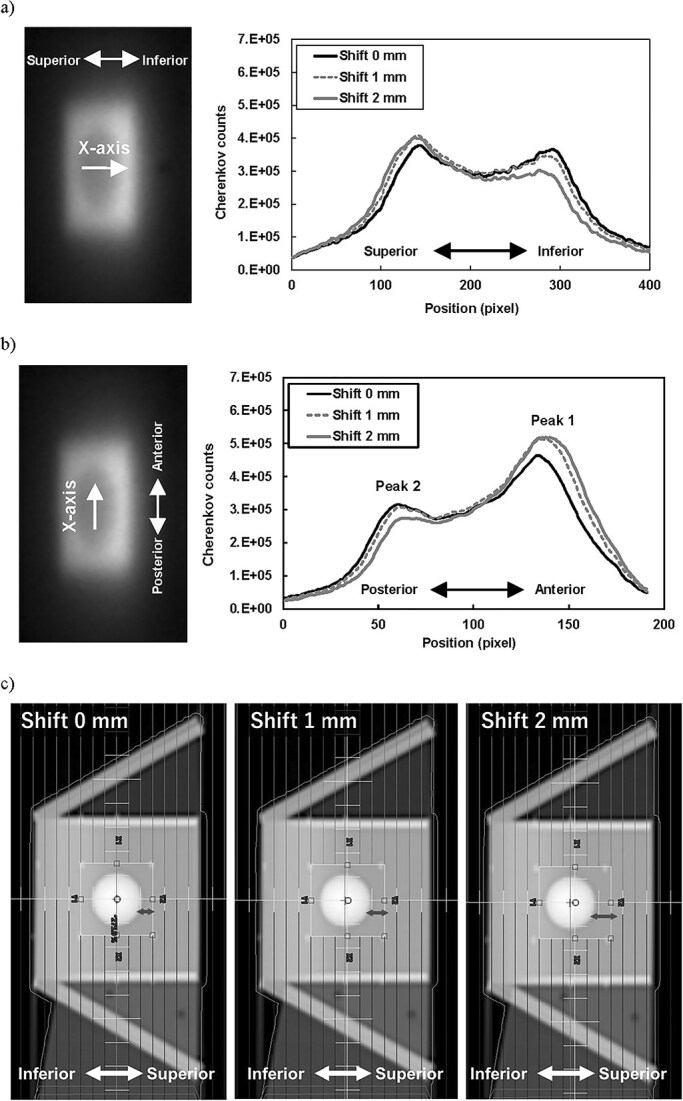
CE profiles at the left side plate of the phantom for the lateral field with displacements of 0, 1, and 2 mm in the (a) inferior and (b) posterior directions. The white arrow indicates the x-axis of the figure, and the pink bar represents the treatment couch. (c) Beam’s eye view of the lateral field.

**Fig. 3:**
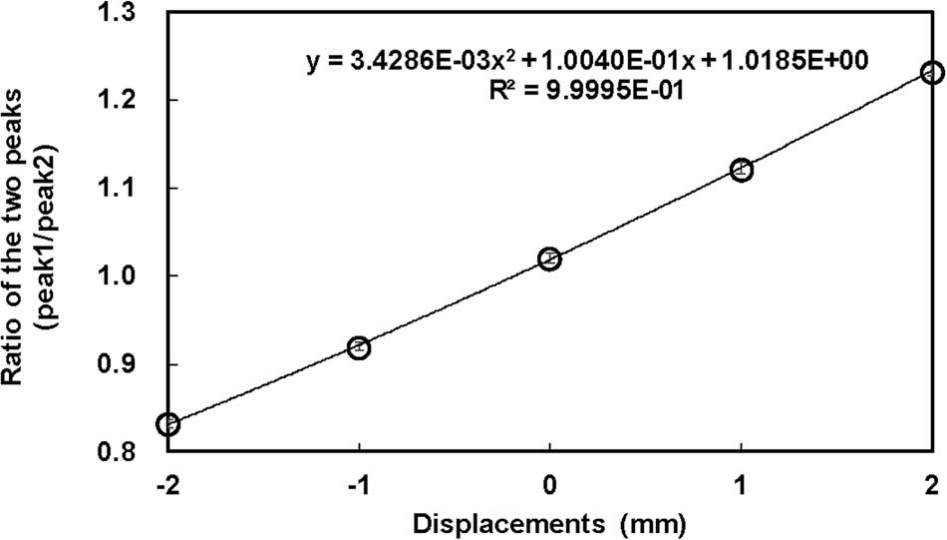
Relationship between predetermined displacements in the anterior–posterior (AP) direction and the ratio of the two peaks (peak 1/peak 2; Fig. 2b) in the CE profiles at the left plate for the lateral field. A second-order polynomial function was used to evaluate the treatment position. Error bars (±0.006) represent variability obtained from the repeatability test.

The background image of the C-QA phantom is shown in Fig. 1b, and the CE observed from the lateral and posterior fields at the four plates is shown in Fig. 1c. These two images (Fig. 1b and c) were obtained simultaneously in a single measurement. Bowl-shaped CE patterns on the anterior and left plates, caused by attenuation from the tungsten target (mock tumor placed in the phantom), were observed. Figure 2a and b present examples of CE profiles with phantom displacements of 0, 1, and 2 mm in specific directions. The two peaks varied depending on the change in the geometric relationship between the beam and the tungsten target. Figure 2c illustrates the beam’s eye view for the lateral field, where the horizontal axis corresponds to the superior–inferior direction. In this figure, the area between the mock tumor and the radiation field in the superior direction, indicated by an arrow, expands as the phantom displacement increases in the inferior direction. Consequently, an increase in radiation intensity is observed in the superior region, corresponding to an increase in CE. This observation is consistent with the results shown in Fig. 2a, where the peak intensity in the superior region exceeds that in the inferior region. As shown in Fig. 2b, when defining the anterior peak as Peak 1 and the posterior peak as Peak 2, the relationship between the Peak1/Peak2 ratio and the displacement demonstrated a comparatively linearity (Fig. 3). A second-order polynomial function was applied to fit the data, showing a strong correlation (correlation coefficient *r* = 0.9992).

Table 1 summarizes the tests for positional treatment accuracy following tumor alignment using CBCT. Both the lateral and posterior fields were used to calculate the SI shifts, and the mean value of the two was reported. The differences in all cases were within 0.5 mm, with the mean and standard deviation across all four tests being 0.1 mm and 0.1 mm, respectively.

**Fig. 4:**
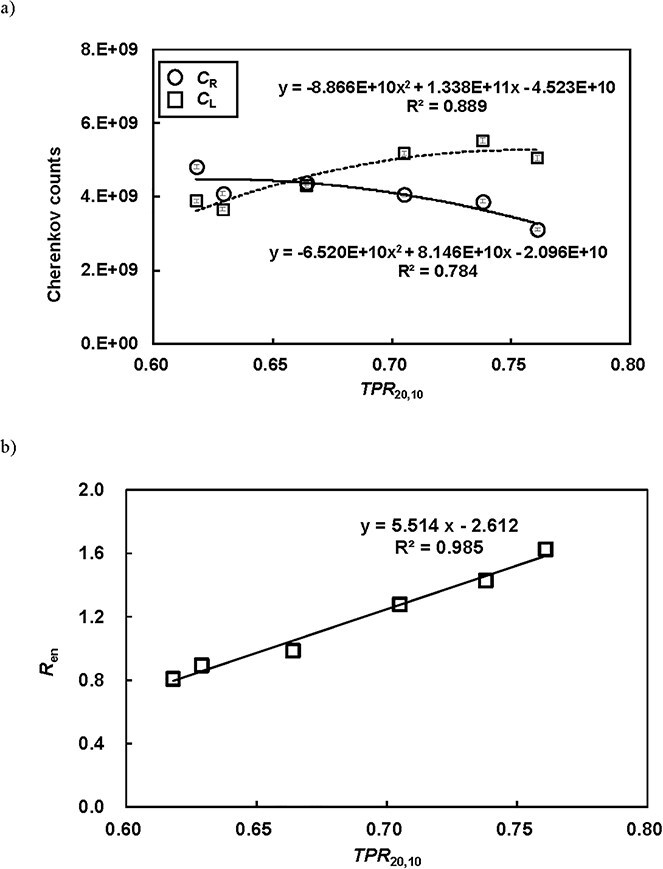
(a) *C*_R_ and *C*_L_ values, and (b) the ratio of CE at the right and left plates (*R*_en_) with respect to *TPR*_20,10_ for all photon energies. Error bars derived from the repeatability test are shown.

Figure 4 shows *C*_R_, *C*_L_, and *R*_en_ with relation to *TPR*₂₀,₁₀ for all photon energies. The three CE energy characteristics exhibited strong correlations and linearity. The best fitting accuracy was observed for *R*_en_, and a second-order polynomial function was applied to the data, as it yielded a higher coefficient of determination than the first-order fit. Additionally, the correlation coefficient was 0.985. Table 2 presents the repeatability (*n* = 5) of positional treatment accuracy using CBCT-based image guidance, gantry angle, *TPR*₂₀,₁₀, and detected Cherenkov counts. The positional accuracy in the SI direction was represented as the mean value derived from the lateral and posterior fields. *TPR*₂₀,₁₀ was calculated using the regression curve obtained for *R*_en_ (Fig. 4b). The mean ± σ of *TPR*₂₀,₁₀ in the RL plate for the repeatability and long-term tests was 0.631 ± 0.004. In comparison, the *TPR*₂₀,₁₀ measured using the PTW Semiflex Ionization Chamber 31 010 for 6X FFF was 0.629, with a difference of 0.001 between the two.

**Table 2:** Statistical analysis of repeatability and long-term measurements of positional treatment accuracy, gantry angle, TPR20,10, and detected counts using CBCT-based image guidance. The five tests were conducted on different days for reproducibility test

	Positional accuracy (mm)			Gantry angle (°)		*TPR*_20,10_ (6XFFF)	Cherenkov counts^b^	X-ray output^c^
	RL	SI^a^	AP	90	180	RL plate	RL plate	
*Reproducibility*								
Mean	0.0	−0.1	0.1	90.1	179.9	0.632	1.028	1.000
σ	0.0	0.0	0.0	0.1	0.1	0.010	0.084	0.004
*Long-term analysis*								
Mean	−0.42	−0.01	0.00	90.1	180.0	0.631	0.998	0.999
σ	0.18	0.14	0.12	0.4	0.4	0.004	0.027	0.003
σ (%)	*NA*	*NA*	*NA*	*NA*	*NA*	0.8%	2.7%	0.3%
Mean + 1.96σ	−0.07	0.28	0.24	90.7	180.9	0.640	1.047	1.005
Mean − 1.96σ	−0.77	−0.31	−0.25	89.3	179.5	0.623	0.952	0.994

^a^The SI direction shows the mean value from that of the lateral and posterior fields.

^b^The Cherenkov counts were normalized at the first measurement. RL: 4.086E + 09 counts and AP: 4.965E + 09 counts for reproducibility test. RL: 1.526E + 09 counts and AP: 1.856E + 09 counts for long-term tests. RL: right–left; SI: superior–inferior; AP: anterior–posterior.

^c^X-ray output measured using the QUICKCHECK webline (Dose calibration was not performed in this term). The X-ray output were normalized at the first measurement.

**Fig. 5:**
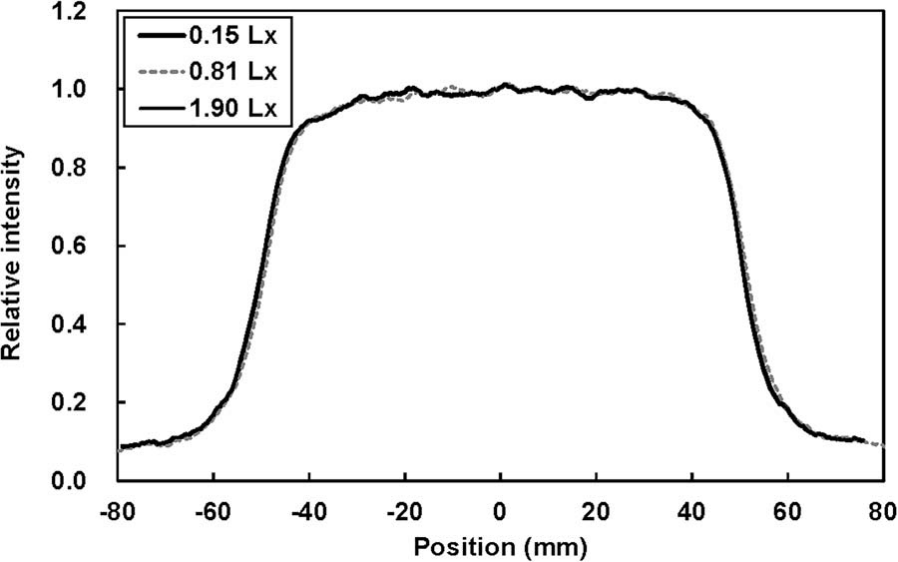
CE profiles obtained under varying ambient light levels in the treatment room.

**Fig. 6:**
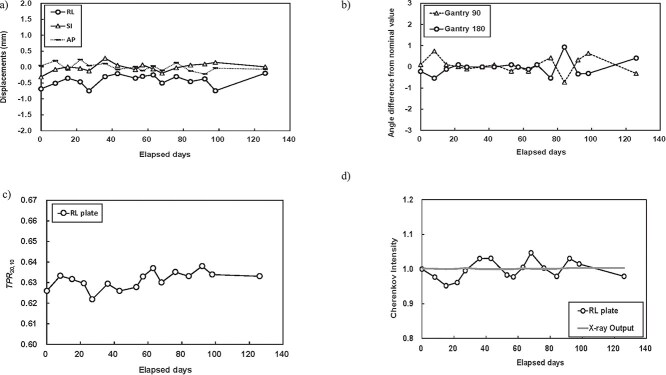
One C-QA test measurement provides assessments of multiple treatment parameters: (a) Positional accuracy using CBCT-based image guidance. (b) Gantry angle deviations from nominal values at 90° and 180°. (c) *TPR*_20,10_. (d) Detected Cherenkov counts and X-ray output measured using the QUICKCHECK webline system (dose calibration not performed during this period). Long-term testing was conducted over three months. RL: right–left, SI: superior–inferior, AP: anterior–posterior.

Lateral CE profiles under three light intensity levels are shown in Fig. 5. The minimum, medium, and maximum light intensities were 0.15, 0.81, and 1.90 lx, respectively. The detected counts per cm^2^ at these intensities were 1.111 × 10^8^, 1.105 × 10^8^, and 1.113 × 10^8^, respectively, with a maximum difference of 0.8%. The noise within a 1 × 1 cm^2^ area (standard deviation) was 1.5%, 1.5%, and 1.8%, respectively, showing no significant change. Long-term treatment parameters, including positional accuracy evaluated via CBCT-based image guidance, gantry angle, *TPR*_20,10_, and detected Cherenkov counts measured with the developed C-QA phantom are presented in Fig. 6, with the corresponding confidence limits summarized in Table 2. The X-ray linear accelerator (linac) output, monitored using the QUICKCHECK webline system, demonstrated a standard deviation of 0.3% over time, which is one-tenth of the deviation observed in the detected Cherenkov counts.

## DISCUSSION

This study introduces a novel, vendor agnostic application of Cherenkov emission (CE) imaging that integrates three routine machine QA tasks: positional accuracy after CBCT-based alignment, gantry angle, and photon energy (*TPR*_20,10_), into a single measurement with rapid automated analysis. To our knowledge, prior reports have not shown a CE-based workflow that (i) directly visualizes the delivered beam on phantom surfaces for end-to-end verification, (ii) extracts all three metrics from one acquisition with rapid automated analysis, and (iii) maintains accuracy comparable to reference devices (*TPR*_20,10_ = 0.631 ± 0.004 vs 0.629 by ion chamber; positional and angular confidence limits approximately ±1 mm and ± 1°). Another contribution is the RL-plate ratio (*R*_en_), which provided strong correlation with *TPR*_20,10_ (*r* ≈ 0.985) and improved robustness to ambient light relative to single-plate counts, enabling energy checks without absolute dosimetry or consumables. In contrast to conventional, multi-device, separately executed workflows such as Winston-Lutz [17] for geometry and ion chamber or film for energy and dose, the proposed CE approach offers an efficient, consumable-free, all-in-one measurement suitable for daily or weekly constancy trending. Limitations include the current emphasis on geometric and energy constancy rather than absolute output, the requirement for a dedicated phantom and camera, and a bottleneck in data transfer and decompression rather than analysis time. These items will be the focus of ongoing work on automation, ambient-light mitigation, multi-center reproducibility, and integration with institutional QA systems.

Recently, Varian’s Machine Performance Check (MPC) has been demonstrated to be reliable and is widely used in clinical practice [18, 19]. It enables easy and efficient execution of comprehensive tests, including geometric and dosimetric checks. Clearly, it is impossible to replace the Varian MPC with our CE-based method. However, our evaluation approach is simple, and measuring Cherenkov emissions from the beam offers an all-in-one measurement for multiple tests. A previous research group reported the use of Cherenkov imaging for daily QA with an MR-linac, introducing an all-in-one method for acquiring and analyzing treatment position accuracy in near-real time [20]. They introduced an all-in-one measurement for acquiring and analyzing treatment position accuracy in near real time using Cherenkov imaging. Similarly, the plastic scintillator-based QA approach is also attractive, and numerous studies have investigated its use for various purposes [21–24].

According to the AAPM Task Group 142 [25, 26], the coincidence between imaging and treatment coordinates should be ≤2 mm for non-SRS/SBRT and ≤ 1 mm for SRS/SBRT. Traditionally, this verification requires two separate tests. In the first, a cubic phantom containing

radiopaque markers is commonly positioned using room lasers as a surrogate for the radiation isocenter. The deviation from the reference CT is then evaluated by registering the reference CT and CBCT images. In the second, the Winston–Lutz test [17] is typically performed. By combining the results of these two tests, using the treatment room lasers as a reference, the coincidence of imaging and treatment coordinates can be verified. Our method was designed to integrate these two tests into a single procedure, using CE to directly assess the delivered radiation field locations after registering the reference CT and CBCT. The procedures and analyses were efficient, simple, and straightforward.

The four tests and long-term evaluations demonstrated excellent results, except for the Cherenkov counts. The confidence limits for positional accuracy and gantry angle were within approximately ±1 mm and ± 1°, respectively. As shown in Fig. 4, the CE on the paired RL plates, *C*_R_ and *C*_L_, depends on photon energy. As photon energy increases, *C*_R_ decreases due to the reduced superficial dose in the build-up region, while *C*_L_ increases because higher-energy photons penetrate more deeply. Consequently, the ratio of CE at the RL plates, denoted as *R*_en_, shows an increasing trend. The calculated *TPR*_20,10_ values in the repeatability and long-term tests were close to the measured *TPR*_20,10_ (0.629). Notably, the coefficient of determination (*R*^2^) for *C*_R_ and *C*_L_ was lower than that for *R*_en_, indicating that the *R*_en_ ratio is more effective for accurately calculating *TPR*_20,10_ than *C*_R_ or *C*_L_ alone. This is because the detected Cherenkov counts can be affected by ambient treatment room light, whereas using the *R*_en_ ratio helps mitigate this influence. These findings allow us to identify photon energy; for example, *TPR*_20,10_ values of 0.618 for 4X and 0.664 for 6X fall outside the confidence interval of 6XFFF calculated by the CE-based measurements, as shown in Table 2. Regarding Cherenkov counts, σ was 2.7%, which is considerably higher than that of a Farmer-type ionization chamber, where σ is typically about 0.05% based on our clinical experience. Figure 5 shows that the relative CE profile shapes are similar under varying ambient lighting conditions. However, the detected Cherenkov counts exhibit a maximum deviation of 0.8%, and σ of the approximately 2% is considered high in this context. Although ambient room light does not directly increase noise or alter the CE profiles, reducing noise in Cherenkov imaging for radiotherapy remains a significant challenge. To address this, higher irradiation MU are often used due to the extremely low CE intensity, or binning techniques are applied to improve signal strength. Regarding the redesign of the C-QA phantom, incorporating a scintillator or a non-attenuating light medium, such as water, at the beam entry plate could enhance emission intensity. These aspects should be further investigated in future work.

This study has several limitations. Our methodology analyzed relative CE data to evaluate positional treatment accuracy via CBCT-based image guidance and *TPR*_20,10_. Specifically, the tests used models based on various predetermined phantom displacements or *TPR*_20,10_ values across all photon energies. However, this approach does not account for potential mechanical errors in couch movement when calculating parameters. Based on our routine QA experience during the study period, we did not observe large couch displacements exceeding 1 mm. Regarding the linac dose output, no method was developed to convert the absorbed dose from the Cherenkov counts. Moreover, considerable variation was observed in the Cherenkov counts, with σ of 2.7%, indicating that the reliability of using Cherenkov signals for absolute dosimetry remains an open issue. AAPM Task Group 198 [25, 26] recommended that the phantom include multiple targets for registration between the reference CT and CBCT during imaging and treatment coordinate coincidence tests. However, our C-QA phantom includes only a single target located at the center. Further studies incorporating multiple targets and irradiation conditions, such as varying gantry and couch angles, are necessary to advance the development of the phantom.

CE-based radiotherapy QA is potentially effective and reliable. Treatment positioning can be directly assessed without the need for conventional dosimetry devices such as film. In our proposed IGRT QA method using CE, a single measurement can evaluate multiple treatment parameters, including positional accuracy via CBCT-based image guidance, gantry angle, and photon energy (*TPR*_20,10_). However, accurately measuring the linac dose output remains a challenge.

## Supplementary Material

Supplementary_Figure_1_rraf072

Supplementary_Figure_2_rraf072
