# Supplementary material for: Cherenkov emission–based quality assurance for linear accelerators
Source: J Radiat Res. 2026 Jan 13;67(1):20–8. doi: 10.1093/jrr/rraf072 (PMC12856039; doi:10.1093/jrr/rraf072)
Supplement: Supplementary_Figure_1_rraf072 [file supplementary_figure_1_rraf072.docx]

| a) |
| --- |
|  |
| b) |
|  |
| Supplementary Figure 1. (a) The developed C-QA phantom. (b) Reference CT images on the axial, coronal, and sagittal planes. Pink color is the treatment couch. The blue arrow indicates a mock tumor. |
