# Supplementary material for: Cherenkov emission–based quality assurance for linear accelerators
Source: J Radiat Res. 2026 Jan 13;67(1):20–8. doi: 10.1093/jrr/rraf072 (PMC12856039; doi:10.1093/jrr/rraf072)
Supplement: Supplementary_Figure_2_rraf072 [file supplementary_figure_2_rraf072.docx]

|  |
| --- |
| Supplementary Figure 2. Method for calculating the gantry angle for the posterior field. It is determined from the displacement from the center of the lower plate ($\Delta x_{lower}$), the displacement from the center of the upper plate ($\Delta x_{upper}$), and the vertical length (*H*) at that location. The same calculation method is applied to the lateral field. |
